# Population Genomic Analysis of *Listeria monocytogenes* From Food Reveals Substrate-Specific Genome Variation

**DOI:** 10.3389/fmicb.2021.620033

**Published:** 2021-02-09

**Authors:** Tyler D. Bechtel, John G. Gibbons

**Affiliations:** ^1^Department of Food Science, University of Massachusetts, Amherst, MA, United States; ^2^Molecular and Cellular Biology Graduate Program, University of Massachusetts, Amherst, MA, United States; ^3^Organismic and Evolutionary Biology Graduate Program, University of Massachusetts, Amherst, MA, United States

**Keywords:** *Listeria*, dairy, microbial genomics, pathogen, food safety

## Abstract

*Listeria monocytogenes* is the major causative agent of the foodborne illness listeriosis. Listeriosis presents as flu-like symptoms in healthy individuals, and can be fatal for children, elderly, pregnant women, and immunocompromised individuals. Estimates suggest that *L. monocytogenes* results in ∼1,600 illnesses and ∼260 deaths annually in the United States. *L. monocytogenes* can survive and persist in a variety of harsh environments, including conditions encountered in production of fermented dairy products such as cheese. For instance, microbial growth is often limited in soft cheese fermentation because of harsh pH, water content, and salt concentrations. However, *L. monocytogenes* has caused a number of deadly listeriosis outbreaks through the contamination of cheese. The purpose of this study was to understand if genetically distinct populations of *L. monocytogenes* are associated with particular foods, including cheese and dairy. To address this goal, we analyzed the population genetic structure of 504 *L. monocytogenes* strains isolated from food with publicly available genome assemblies. We identified 10 genetically distinct populations spanning *L. monocytogenes* lineages 1, II, and III and serotypes 1/2a, 1/2b, 1/2c, 4b, and 4c. We observed an overrepresentation of isolates from specific populations with cheese (population 2), fruit/vegetable (population 2), seafood (populations 5, 8 and 9) and meat (population 10). We used the Large Scale Blast Score Ratio pipeline and Roary to identify genes unique to population 1 and population 2 in comparison with all other populations, and screened for the presence of antimicrobial resistance genes and virulence genes across all isolates. We identified > 40 genes that were present at high frequency in population 1 and population 2 and absent in most other isolates. Many of these genes encoded for transcription factors, and cell surface anchored proteins. Additionally, we found that the virulence genes *aut* and *ami* were entirely or partially deleted in population 2. These results indicate that some *L. monocytogenes* populations may exhibit associations with particular foods, including cheese, and that gene content may contribute to this pattern.

## Introduction

*Listeria monocytogenes* is a gram positive, foodborne pathogen that causes the severe infection listeriosis (Buchanan et al., 2017). Listeriosis infects an estimated 1,600 people and causes about 260 deaths annually in the United States^1^. Although listeriosis is relatively rare, it is regarded as a serious public health concern due to its high mortality rate (20–30%) (Choi et al., 2018). Additionally, infants, the elderly, and immunocompromised individuals are at an increased risk of severe cases of listeriosis, which can result in symptoms such as meningitis and septicemia (Jacquet et al., 2004). *L. monocytogenes* infections can also threaten pregnant women because the pathogen possesses the unique ability to permeate the placental wall and infect the fetus which can lead to miscarriage or stillbirth (Cotter et al., 2008; Hilliard et al., 2018).

*L. monocytogenes* is ubiquitous in the environment as a saprophyte, and is abundant in soil and water (Smith et al., 2018). The wide distribution of *L. monocytogenes* in the environment is due to its ability to persist under extreme conditions including cold temperatures, dry conditions, low pH, and high salt environments (Magalhães et al., 2016; Hingston et al., 2017). The ecological range of this species in addition to its ability to form biofilms enables *L. monocytogenes* to contaminate and persist on a variety of substrates. These characteristics result in *L. monocytogenes* frequent contamination of different foods including dairy, meat, seafood, and fresh produce (Jalali and Abedi, 2008; Shamloo et al., 2019). A recent outbreak linked to a mushroom supplier in Korea resulted in 47 illnesses and 4 deaths over the last 3 years across five countries (Centers for Disease Control and Prevention, 2020; Food Safety News, 2020). Another outbreak occurred between February and June of 2020, when six illnesses and two deaths were reported in the Netherlands from contaminated smoked trout filets (Food Safety News, 2020).

There are 13 known serotypes of *L. monocytogenes* that fall into four major phylogenetic lineages (Orsi et al., 2011). Lineage I and II are commonly found in food, human outbreak cases, and in the environment, while lineages III and IV are primarily isolated from ruminant animals (Orsi et al., 2011). Lineage I has been associated with epidemic listeriosis cases while lineage II is has been associated with isolates sourced from food and food environments (Nightingale et al., 2005, 2006, 2008; Orsi et al., 2011). This trend suggests that genetic variation between the two lineages results in lineage I’s ability to infect humans more efficiently, and lineage II’s ability to colonize food substrates. Additionally, lineage II isolates have reduced virulence compared to lineage I (Jacquet et al., 2004; Nightingale et al., 2005). This reduction in virulence may, in part, be due to the observation that over 30% of lineage II isolates contain a premature stop codon in *inlA*, a gene encoding Internalin A, a key virulence factor required for the invasion of epithelial cells (Nightingale et al., 2005; Bonazzi et al., 2009).

*L. monocytogenes* often occurs in ready-to-eat foods that require minimal heating or cooking, such as cheeses, and other fermented dairy products. There are several possible routes *L. monocytogenes* can enter and contaminate dairy, including transmission from infected ruminants to milk, and through improper pasteurization or the usage of contaminated equipment during post-processing (Melo et al., 2015). The production process and physical attributes of cheese make it a suitable substrate for *L. monocytogenes* growth. The pH range, water content, and salt concentrations of soft cheeses are often inhibitory to other microorganisms, but suitable for *L. monocytogenes* (Melo et al., 2015; Hingston et al., 2017). A survey of 374 European red-smear cheeses revealed 6.4% of cheeses were contaminated with *L. monocytogenes* (Rudolf and Scherer, 2001). In the United States since 2011, a number of multistate foodborne *L. monocytogenes* outbreaks were the result of contaminated cheese, raw milk, and ice cream^2^.

The objective of this study was to investigate the association between population structure and the food source from which *L. monocytogenes* strains were isolated. We analyzed the genomes of hundreds of *L. monocytogenes* strains from food, and identified 10 major populations. Specific populations showed associations with cheese, fruit/vegetables, seafood and meat. Using comparative and population genomic analysis, we identified genes that were present at greater frequency in the population associated with cheese and a closely related population. Our results shed light on potential candidate genes involved in the specialization to particular food substrates.

## Materials and Methods

### Whole-Genome Data

We were specifically interested in addressing whether *L. monocytogenes* serotypes display associations with food types, with particular interest in strains isolated from dairy and cheese. To compile a diverse collection of genomes from meat, seafood, dairy, and fruit/vegetables, we used the NCBI Pathogen Detection^3^ database. We downloaded whole genome fasta files of 1,213 strains isolated from “cheese”, “drain raw meat”, “seafood processing environment”, “hass avocados”, “ice cream”, “lettuce”, “milk”, “plain cream cheese spread”, “potato”, “raw meat”, “raw milk”, “raw cut vegetables”, “retail meat”, “salami”, “shrimp”, “slaughterhouse environment”, and “smoked salmon” on 02/24/2020 (Supplementary Table S1). The non-clonally identical isolates of *L. monocytogenes* originated from 19 countries (Canada = 24, United States = 272, Mexico = 10, Brazil = 2, Chile = 12, Uruguay = 5, Denmark = 1, France = 7, Germany = 1, Greece = 1, Ireland = 3, Italy = 64, Norway = 3, Poland = 2, Switzerland = 10, United Kingdom = 62, South Korea = 1, Australia = 1 and New Zealand = 19) and six major geographic regions (North America (NA) = 296, Central America (CA) = 10, South America (SA) = 19, Europe (EU) = 154, Asia (AS) = 1, and Oceania (OC) = 20). To assess genome assembly quality, we used BUSCO v3.1.0 to quantify the percentage of complete single copy orthologs present in each *L. monocytogenes* genome using the “bacillales_odb9” dataset^4^ (Simão et al., 2015).

### Relationship of *L. monocytogenes* Isolates

We initially examined the relationship of the *L. monocytogenes* isolates using an alignment of 1,92,465 polymorphic sites across the 1,213 genomes. SNPs were extracted from whole genome assemblies using the Phylogenetic and Molecular Evolution (PhaME) analysis tool v1.0.2 with default settings (Ahmed et al., 2015), using *L. monocytogenes* serotype 4b str. F2365 as the reference (Nelson et al., 2004). We then identified and removed all but one occurrence of isolates with identical genotypes (i.e., isolates with zero SNP differences were considered clones). After this clonal-correction, we removed sites where minor allele frequency was <0.5%. Our final dataset consisted of 504 isolates and 66,698 SNPs. We used Principal Component Analysis (PCA) to investigate the relationship between the *L. monocytogenes* isolates. PCA was performed in TASSEL with eigenvalue decomposition on the covariance matrix (Bradbury et al., 2007). Additionally, we used RheirBAPS (Tonkin-Hill et al., 2018) and Discriminant analysis of principal components (DAPC) (Jombart et al., 2010) to further analyze population structure, using a subset of 661 SNPs that were spaced evenly throughout the genome. In RheirBAPS we used the parameters max.depth = 2, n.pops = 100, n.extra.rounds = Inf, and assignment.probs = TRUE. RheirBAPS predicts the most likely population number given two levels of clustering (max.depth argument), for which we considered the first level of clustering the optimal population number. For DAPC, the number of distinct populations was predicted using the “find.clusters” *k*-means clustering algorithm and by calculating the Bayesian Information Criterion (BIC) value for each *K* between?1 and 100. Predicting the optimal population number is often unclear and complex in panmictic natural populations, and we considered the optimal population number as the first local BIC minimum. We evaluated the population assignments between RheirBAPS and DAPC with the PCA patterns to define 10 “consensus populations”. From here forward, we describe populations 1–10 as P1, P2, P3, P4, P5, P6, P7, P8, P9, and P10.

Lastly, we constructed an approximately maximum likelihood tree from the alignment of 66,698 SNPs using FastTree2 (Price et al., 2010) with 100 bootstrap replicates. For visualization and annotation of the phylogenetic tree, the R packages “ggtree”, “ggplot2”, and “APE” (Wickham, 2016; Yu et al., 2017; Paradis and Schliep, 2019). These packages were used to produce a tree that displays lineage, serovar and population structure assignments. To better visualize these characteristics, the genetic clusters and various serovars were differentially color coded using a custom color palette created in RColorBrewer using the hexadecimal color picking tool from the (Html Color Picker, 2020).

### Genomic Prediction of *L. monocytogenes* Serotypes

In order to assign isolates to major *L. monocytogenes* serotypes, we ran PhaME v1.0.2 again with the 504 isolates and an additional 166 *L. monocytogenes* isolates of known serotypes from Hingston et al. (2017). The genomes from the Hingston et al. (2017) isolates were obtained from NCBI Bioproject PRJNA329415. The *L. monocytogenes* EGD-e reference genome was used as the reference genome in PhaME and was acquired from the NCBI database under RefSeq accession number NC_003210.1. We used PCA to infer the relationships between individuals as described above, and we constructed an approximately-maximum-likelihood phylogenetic tree using FastTree v2.1.10 with 100 bootstrap replicates (Price et al., 2010). Serotyping was inferred from the proximity of each isolate to the isolates with known serotypes in the phylogenetic tree and PCA plot. The 166 additional isolates and EGD-e reference genome were only used for serotyping and were not included in subsequent analyses.

### Overrepresentation of Isolates by Population, Geographical Origin, Serotype and Food Source

We used a *χ*^2^ test of independence to test the null hypotheses that variables were randomly distributed between each other (population assignment, geographical origin, serotype, and food source). We considered *χ*^2^ standardized residuals ≥2 and ≤−2 as indicative of an overrepresentation and underrepresentation, respectively, of a particular category. These values represent two standard deviations from the mean. Statistical analysis was conducted in R (R Core Team, 2017).

### Identification of Lineage Specific Genes

**FIGURE 1:**
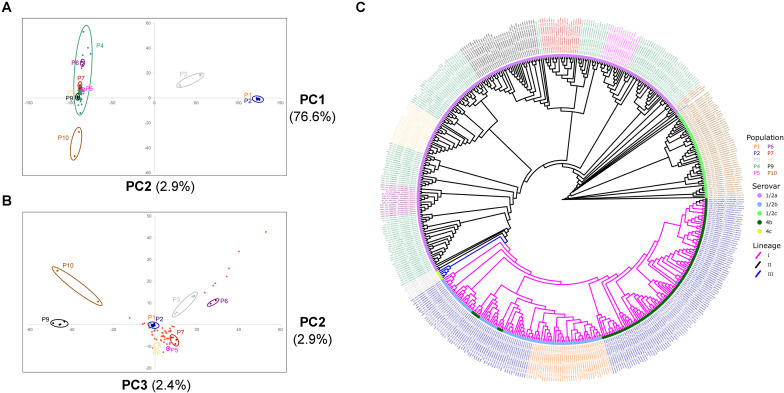
Population structure of 504 *L. monocytogenes* strains isolated from food. Principal component analysis (PCA) of 66,698 SNPs for PC1 and PC2 **(A)** and PC2 and PC3 **(B)**. Percent of variance explained by each PC is reported in parentheses. Isolates are colored and circled based on the consensus population assignment predicted through hierBAPS and DAPC (Supplementary Figure S2). P4 is not circled in the PC2 vs. PC3 plot for clarity. **(C)** FastTree2 approximately maximum likelihood phylogenetic tree inferred from the SNP alignment generated by PhaME. Taxon labels, external nodes, and branches are colored according to population assignment, serovar, and lineage, respectively.

**FIGURE 2:**
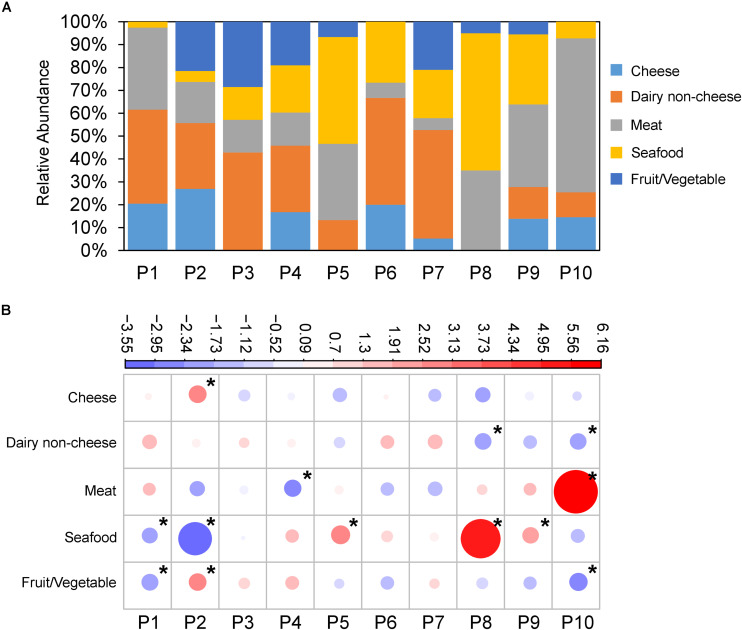
*L. monocytogenes* populations are associated with food source. **(A)** Relative abundance of food source *L. monocytogenes* were isolated from (*Y*-axis) across populations (*X*-axis). **(B)** Overrepresentation and underrepresentation of isolates from populations versus food type. Plots represent standardized residuals from χ^2^ analysis. Circle size represents absolute value of the standardized residual, while color represents positive (red) and negative (blue) values. Stars indicate standardized residuals with absolute values ≥2 (two standard deviations). P2 was overrepresented with strains isolated from cheese and fruit/vegetables, P5, P8, and P9 with seafood, and P10 with meat.

Because P2 and P1 are so closely related and because P2 displayed an overrepresentation for cheese (Figures 1, 2), we identified genes specific to P2 and genes specific to P1 + P2 using two different approaches. First, we used the LS-BSR pipeline (Sahl et al., 2014) to identify genes present in all isolates (P2, or P1 + P2) and absent in all other isolates by requiring BSR values ≥0.8 for gene presence in all isolates, and BSR values ≤0.4 for gene absence in all remaining isolates. Specifically, we used the “compare.py” command within LS-BSR to identify genes specific to these particular groups of isolates, with the BSR matrix and a FASTA file of all coding sequences across samples as inputs. We also used a less stringent approach by identifying all genes with an average BSR value ≥0.9 in P2 or P1 + P2, and an average BSR value ≤0.2 in all other isolates. This approach allows for some variation in gene presence/absence within populations. Second, we used the Roary pipeline to identify genes specific to P2 and P1 + P2 (Page et al., 2015). In this approach, we first used Prokka to annotate each of the 504 *L. monocytogenes* genomes using “−−kingdom Bacteria” and default settings (Seemann, 2014). Next, we used Roary to generate a pan-genome from the Prokka gff files, and used MAFFT (−e and −n options) to generate a core gene alignment (Katoh and Standley, 2013). Finally, we used the “gene_presence_absence.Rtab” output file to determine the presence and absence patterns of genes in P1 and P1 + P2 versus all other populations.

### Antimicrobial Resistance and Virulence Gene Typing

We investigated the gene presence/absence patterns of 30 previously characterized antimicrobial resistance genes (Granier et al., 2011; McMillan et al., 2019) across the 10 *L. monocytogenes* populations. We obtained sequences for each gene from the Comprehensive Antibiotic Resistance Database (McArthur et al., 2013). To investigate the distribution of virulence genes across the genetic clusters, a library of 92 *L. monocytogenes* virulence genes were obtained from the virulencefinder database (Joensen et al., 2014). The set of antibiotic resistance genes and virulence genes were independently used as a reference with the “gene screen” method implemented in LS-BSR using the default parameters.

## Results

### Quality Assessment of *L. monocytogenes* Genome Assemblies

To assess the quality of genome assemblies, we used BUSCO to quantify the percentage of complete Bacillales single copy orthologs found in each of the 504 *L. monocytogenes* isolates analyzed (Simão et al., 2015). The average and median complete BUSCO gene presence percentages were 96.58 and 96.9%, respectively (minimum = 90.3 and maximum = 97%), and 97% of isolates had ≥95% recovery of complete BUSCO genes (Supplementary Table S1). These results suggest that the vast majority of analyzed genome assemblies are high quality.

### Population Structure of *Listeria monocytogenes* Isolates From Food

Using a number of approaches, we inferred the population structure of 504 non-clonally identical isolates of *L. monocytogenes* from food. First, we used the Bayesian Analysis of Population Structure (BAPS) software (Jombart et al., 2010) to predict population structure using a subset of 661 SNPs that were evenly spaced across the genome to minimize physical linkage between markers. BAPS analysis suggested the presence of 12 populations. Next, we used DAPC with the 661 SNP marker set to predict population structure (Jombart et al., 2010). With DAPC, we observed a local minimum BIC value at *K* = 12, before BIC increased at *K* = 13 and *K* = 14 then decreased again at *K* = 15 (Supplementary Figure S1). For this reason, we chose to analyze DAPC population structure at *K* = 12. Individual population assignment between BAPS and DAPC were mostly agreeable with a few exceptions: (1) BAPS population 2 was divided into three DAPC populations (5, 6, and 12), (2) BAPS populations 3 and 4 were combined into DAPC population 1, and (3) BAPS populations 5 and 6 were made up of DAPC populations 7, 8, and 11 (Supplementary Figure S2). Next, we performed PCA on the entire set of 66,698 SNPs to visualize population structure. We evaluated the BAPS, DAPC, and PCA population structure to define 10 consensus populations (P1−P10) (Figures 1A,B and Supplementary Figure S2). PC1 explained 76.6% of variance and separated populations P1, P2, P3, and P6 (Figure 1A), PC2 explained 2.9% of variance and separated populations P4, P5, P6, and P7 (Figures 1B,C), and PC3 explained 2.4% of variance and separated population P9 (Figure 1B).

Additionally, we constructed an approximately-maximum likelihood phylogenetic trees using FastTree2 (Price et al., 2010) with the 504 isolates and the *L. monocytogenes* serotype 4b str. F2365 reference genome, and with the 504 isolates and 166 previously sequenced and serotyped isolates (Hingston et al., 2017) in order to bioinformatically predict serovar and lineage for each isolate. Importantly, the phylogenetic results are in strong agreement with the population structure results (Figure 1). All but two of the 504 isolates were assigned with confidence to a serovar based on their phylogenetic proximity to the isolates that were previously serotyped (Figure 1C and Supplementary Figure S3). The two exceptions were isolates MEAT_GCA_004724945_1_PDT000123584_4_genomic and VEG_GCA_004726465_1_PDT000272419_3_genomic which were assigned to serovar 1/2a. We identified three lineages and five distinct serovars within the 504 isolates. Lineage I is exclusive to P1 and P2 which consists of 206 isolates from serotypes 4b and 1/2b. P3 consists of seven isolates from lineages II and III, and serovars 1/2a and 4c. The remaining populations were made up of lineage II isolates. P4-9 consist of 236 isolates of serovar 1/2a. P10 consists of 51 isolates from serovar 1/2c and 4 isolates from serovar 1/2a. The low occurrence of lineage III isolates and complete absence of lineage IV isolates is in line with the observation that these two lineages are rarely isolated from food-associated environments (Orsi et al., 2011).

### Overrepresentation of Populations From Geographic Origin, Serotype, and Food Source

Next, we evaluated whether isolates from each population were associated with geographical origin (North America (NA), Central America (CA), South America (SA), Europe (EU), Asia (AS), and Oceania (OC)) or any of the major food sources (cheese, dairy non-cheese, meat, seafood, and fruit/vegetable). We rejected the null hypothesis that populations were uniformly distributed across the six geographic regions (*X*^2^ = 151.37, df = 45, *p-*value < 2e-13). P1 was overrepresented with isolates from NA and underrepresented with isolates from EU, P2 was overrepresented with isolates from SA and underrepresented with isolates from OC, P4 was underrepresentated with isolates from SA, P7 was overrepresentated with isolates from CA, P8 and P9 were overrepresentated with isolates from OC and underrepresentated with isolates from OC, and P10 was overrepresented with isolates from EU and underrepresentated with isolates from NA (Supplementary Figure S4).

Additionally, we rejected the null hypothesis that isolates from the 10 populations (P1–P10) were evenly distributed across food sources (*X*^2^ = 190.16, df = 36, *p-*value < 2.2e-16) (Figure 2A). P2 showed an overrepresentation of isolates from cheese and fruit/vegetable, P5, P8, and P9 showed overrepresentations of isolates from seafood, and P10 showed an overrepresentation of isolates from meat (Figure 2B).

We additionally tested the null hypothesis that serovars are evenly distributed across food types. Again, the null hypothesis was rejected (*X*^2^ = 135.49, df = 16, *p* < 2.2e-16) indicating that there is a nonrandom distribution of serovars amongst the food sources. Serovar 1/2a was overrepresented in seafood, 1/2b was overrepresented in dairy non-cheese, 1/2c was overrepresented in meat, and 4b was overrepresented in cheese (Supplementary Figure S5).

### Genes Unique to Genetic Clusters Associated With Dairy

**FIGURE 3:**
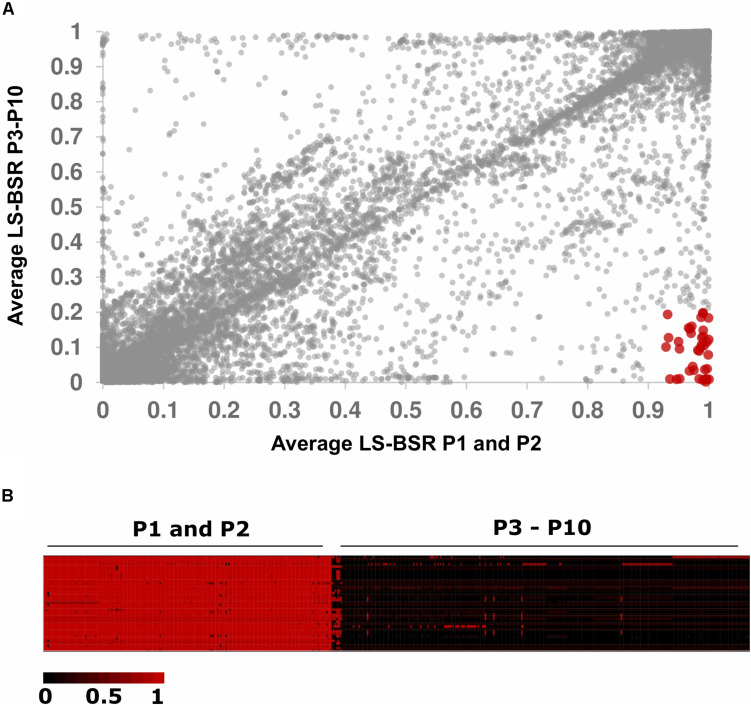
Genes unique to populations 1 and 2. **(A)** Dot plot of average BSR values for P1 and P2 (*X*-axis) versus P3–P10 (*Y*-axis). Forty genes had BSR scores ≥0.90 in P1 and P2 and BSR scores ≤0.20 in P3–P10 (shown in red). **(B)** Heat map of BSR scores for the 40 genes uniquely present in P1 and P2 compared to P3–P10. Rows represent genes, and columns represent individuals.

We sought to identify genes unique to P2 because this population displayed an overrepresentation of strains isolated from cheese (Figure 2). We used LS-BSR and Roary to identify genes present in P2 and absent in all other populations (Sahl et al., 2014; Page et al., 2015). Using LS-BSR and Roary, we identified zero genes and one gene, respectively, that were uniquely present in P2 and absent in all other populations. The gene identified by Roary was annotated as *cobD*, which encodes a protein with L-Threonine-O-3-phosphate Decarboxylase activity that is involved in cobalamin biosynthesis (Fang et al., 2017). Because P1 and P2 are so closely related (Figure 1), we repeated our analysis by comparing the combined gene content of P1 and P2 versus all other isolates. This analysis yielded 4 genes with LS-BSR and 51 genes with Roary that were uniquely present in all P1 and P2 genomes but absent in all other isolates. Three of genes identified with LS-BSR encode predicted TetR/AcrR family transcriptional regulators, and the forth gene encodes a protein that contains a LPXTG cell wall-anchoring domain that is commonly found in pathogenic strains of *Listeria* (Reis et al., 2010). Because the LS-BSR analysis is stringent in requiring that all isolates in a population have BSR values meeting presence/absence cut-offs, we also examined genes with average BSR scores ≥ 0.90 in P1 and P2 and average BSR scores ≤0.20 in P3–P10 (Figure 3A). Using these less stringent, but still conservative cut-offs, we identified 40 genes that were present in the majority of P1 and P2 isolates and absent in the majority of P3-P10 isolates (Figure 3B and Supplementary Table S2). These genes include significant BLAST hits to Crp/Fnr family transcriptional regulators, GntR family transcriptional regulators, TetR/AcrR family transcriptional regulators, GNAT family *N*-acetyltransferases, a methyltransferase, an *N*-acetylmuramic acid 6-phosphate etherase, genes encoding cell wall proteins containing the LPxTG motif, and genes encoding proteins with the MucBP mucin-binding domains (Figure 3 and Supplementary Table S3). Using Roary, we identified 51 genes unique to P1 and P2 but absent in all other populations. This collection of genes was highly similar to those identified using the less conservative parameters with LS-BSR (Supplementary Table S3).

### Antimicrobial Resistance and Virulence Gene Profiles of *Listeria monocytogenes* Isolates

**FIGURE 4:**
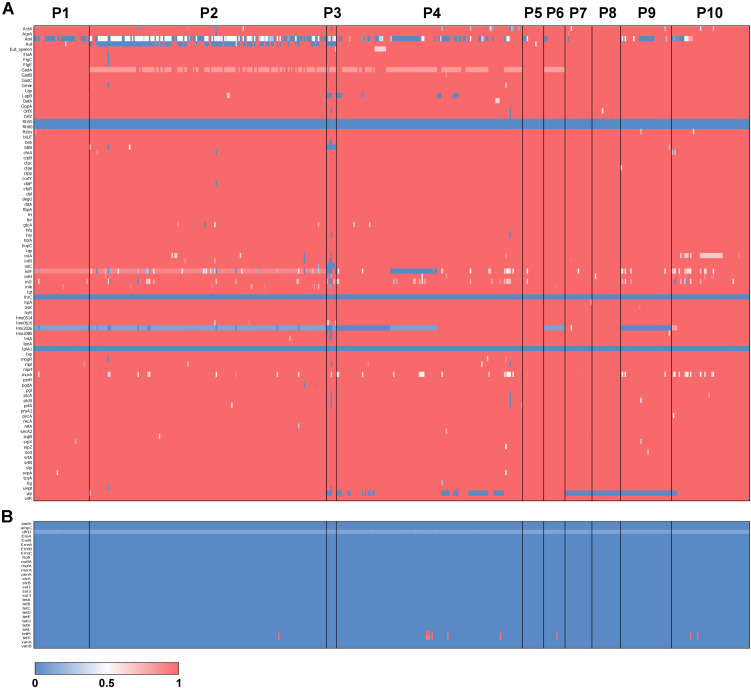
Antimicrobial resistance and virulence gene typing across *L. monocytogenes.* Heatmaps of BSR scores for 90 *L. monocytogenes* virulence genes **(A)** and 30 antimicrobial resistance genes **(B)**. Rows represent genes, and columns represent individuals with populations labeled.

To understand the prevalence of antimicrobial resistance genes and virulence genes across the 504 isolates, we used the gene-screen method in LS-BSR to characterize the presence/absence patterns of 30 antimicrobial resistance genes and 92 *L. monocytogenes* virulence genes. The results of this analysis suggest that the vast majority of antimicrobial resistance and virulence genes do not have an association with a particular population (Figure 4). However, several virulence genes show population specific patterns of presence/absence. For instance, *vip*, a cell surface protein required for entry into some mammalian cells (Cabanes et al., 2005) was absent in P7, P8, P9, and some isolates of P4. *lmo2026* encodes a class I internalin that is involved in adhesion and colonization of host tissue (Popowska et al., 2017) and BSR scores suggest this gene is partially deleted in P1, P2, P3, P5, P9, and ∼60% of P4 isolates. *btlB* plays a role in intestinal persistence (Begley et al., 2005) and is absent from P3, but present in almost all other isolates. *aut*, which encodes an autolytic protein necessary for cell invasion (Cabanes et al., 2004), is variably absent within P2 but present in the vast majority of other isolates. Finally, *ami*, which also encodes for an autolysin (Milohanic et al., 2004), is variably absent in P1, P2, P3, P4, P9, and P10, and mostly present in P5, P6, P7, and P8. Of the 30 antimicrobial resistance genes surveyed, only *tetM* and *tetS* were detected. These genes are involved in tetracycline resistance (Charpentier and Courvalin, 1999) and were present in only 10 isolates (∼2% of isolates) and spread across four populations (P2 = 1, P4 = 6, P6 = 1 and P10 = 2) (Figure 4B).

## Discussion

We analyzed the population genomics of 504 *L. monocytogenes* isolates from the food environment and identified 10 distinct populations spanning isolates from lineages I, II and III and serovars 1/2a, 1/2b, 1/2c, 4b, and 4c (Figure 1). The low occurrence of lineage III isolates and complete absence of lineage IV isolates is in line with the observation that these two lineages are rarely isolated from food-associated environments (Nightingale et al., 2008; Orsi et al., 2011). PC1 and PC2 explain a large portion of observed variation (81.9%), and the remaining variation can potentially be attributed to recombination by horizontal gene transfer (HGT). Though natural transformation has not been directly observed in *L. monocytogenes* (Borezee et al., 2000) the genome contains homologues for the competence machinery encoding genes (*ComG*, *ComEA*, *ComEC*, and *ComFA*) (Claverys and Martin, 2003; Chen and Dubnau, 2004), suggesting the potential for this mechanism of HGT. Additionally, hundreds of *L. monocytogenes* bacteriophages have been identified and several are capable of generalized transduction (Chen and Novick, 2009; Upham et al., 2019). Lastly, a number of studies have demonstrated that *L. monocytogenes* can receive genetic information through conjugative transfer (Poyart-Salmeron et al., 1992; Charpentier and Courvalin, 1999; White et al., 2002; Bertsch et al., 2013). At the population level, we observed several associations between populations and particular food sources (Figure 2). Here, we focus primarily on P2, which is composed of lineage I isolates, because this population displayed an overrepresented of isolates from cheese.

Between the 10 populations, we identified population specific gene presence/absence patterns for several key virulence genes (Figure 4). Among these genes was *vip*, which was absent in P7, P8, P9 and some isolates of P4. *vip* encodes a LPXTG protein that utilizes the host Gp96 endoplasmic reticulum receptor to gain entry into the mammalian cell (Cabanes et al., 2005). Additionally, *aut* was variably absent from P2 while the gene was primarily present in all other populations (Figure 4A). *aut* is a cell surface autolysin that is required for entry into eukaryotic cells (Cabanes et al., 2004). In line with our observations, a study of 121 *L. monocytogenes* isolates revealed that 60% of lineage I isolates contained the *aut* gene compared to 98% in lineage II (Kim et al., 2018). Lastly, almost all isolates of P2 possessed intermediate BSR scores for *ami*, an autolysin-encoding gene, suggesting a partial deletion (Figure 4A). *ami* knockouts displayed significantly less adhesion to eukaryotic cells compared to the wild-type, indicating *ami* functions in the attachment to host cells (Milohanic et al., 2004).

P2 was comprised of isolates from Lineage I, which was also the most common lineage isolated from milk, milk filters, and milking equipment collected from bovine dairy farms in the United States over a 12-year period (Kim et al., 2018). Further, an assessment of hazards associated with the spread of *L. monocytogenes* in Switzerland over 10 years, observed a significant positive association between serotype 1/2b isolates with hard and semi-hard cheeses (Pak et al., 2002). Finally, 91% of *L. monocytogenes* strains isolated from more than 4,000 dairy samples from England and Wales were from serotype 1/2 (58%) and 4b (33%) (Greenwood et al., 1991). Cheese population P2 was comprised of 33% of isolates from serovar 1/2b and 67% from serovar 4b.

Despite the overrepresentation of cheese in P2 and dairy in P1 and P2 combined, we identified few genes that were present in high frequency in P1 and P2 and low frequency or absent in all other isolates (Figure 3). P1 and P2 are very closely related (as indicated by the lack of separation between P1 and P2 by PC1, PC2, or PC3 in Figures 1A,B). Interestingly, Doumith et al. (2004) identified five serovar 4b-specific genes in their investigation of lineage-specific genes between epidemic serovar 4b and a non-epidemic serovar 1/2a. Two of these five genes were transcriptional regulators, while the remaining three genes encoded LPXTG anchoring proteins. Similarly, we identified four genes that were present in all P1 and P2 isolates and absent in all other isolates. Three of these genes encode transcriptional regulators while the other encodes a LPXTG cell wall-anchoring protein. Using a less stringent approach, we identified > 40 genes with high gene presence frequency in P1 and P2 that were present in low frequency in P3–P10 (Figure 3 and Supplementary Tables S2, S3). These genes also contained transcription factors and cell surface LPXTG encoding genes. Proteins containing leucine rich repeat (LRR) and LPXTG domains function in the attachment and invasion of host cells (Kuenne et al., 2013). It is important to note that other mutations, such as short indels and SNPs, can also contribute to genomic and phenotypic divergence between populations. For instance, a number of mutations cause a premature stop codon variant in the *inlA* internalin encoding gene, which results in a truncated protein that is secreted instead of anchored to the cell wall (Nightingale et al., 2005, 2006, 2008). Our results suggest that particular *L. monocyotogenes* genotypes may be associated with the colonization of and persistence in certain food environments, such as dairy and cheese (Figure 2). To further address this hypothesis, physiological and biochemical assays as well as functional analysis of candidate genes in the fermented dairy environment will be required.

## Data Availability Statement

The original contributions presented in the study are included in the article/Supplementary Material, further inquiries can be directed to the corresponding author/s.

## Author Contributions

TB and JG designed the study, conducted the analysis, and wrote the manuscript. Both authors contributed to the article and approved the submitted version.

## Conflict of Interest

The authors declare that the research was conducted in the absence of any commercial or financial relationships that could be construed as a potential conflict of interest.

## Footnotes

1 https://www.cdc.gov/listeria/index.html

2 https://www.cdc.gov/listeria/outbreaks/index.html

3 https://www.ncbi.nlm.nih.gov/pathogens/

4 https://busco-data.ezlab.org/v4/data/lineages/
