# Supplementary material for: Population Genomic Analysis of Listeria monocytogenes From Food Reveals Substrate-Specific Genome Variation
Source: Front Microbiol. 2021 Feb 9;12:620033. doi: 10.3389/fmicb.2021.620033 (PMC7902062; doi:10.3389/fmicb.2021.620033)
Supplement: Supplementary file 1 [file Image_1.PDF]

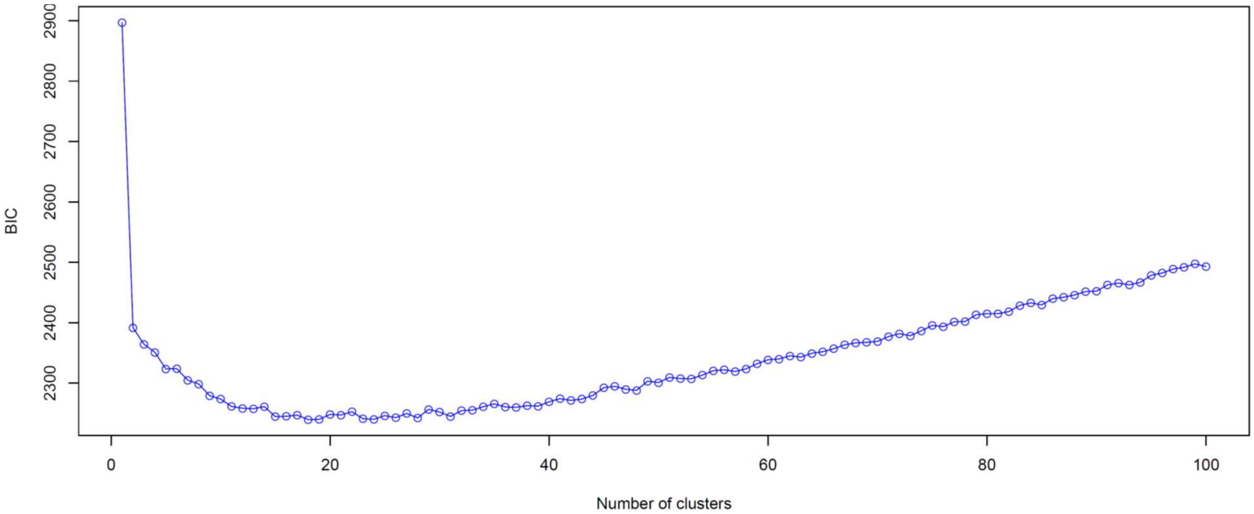

Supplementary Figure S1. Bayesian Information Criterion values (Y-axis) across DAPC population number (X-axis). BIC values were calculated for *k*=1-100. *k*=12 corresponds to the first local minimum BIC valley.
