# Supplementary material for: Population Genomic Analysis of Listeria monocytogenes From Food Reveals Substrate-Specific Genome Variation
Source: Front Microbiol. 2021 Feb 9;12:620033. doi: 10.3389/fmicb.2021.620033 (PMC7902062; doi:10.3389/fmicb.2021.620033)
Supplement: Supplementary file 2 [file Image_2.PDF]

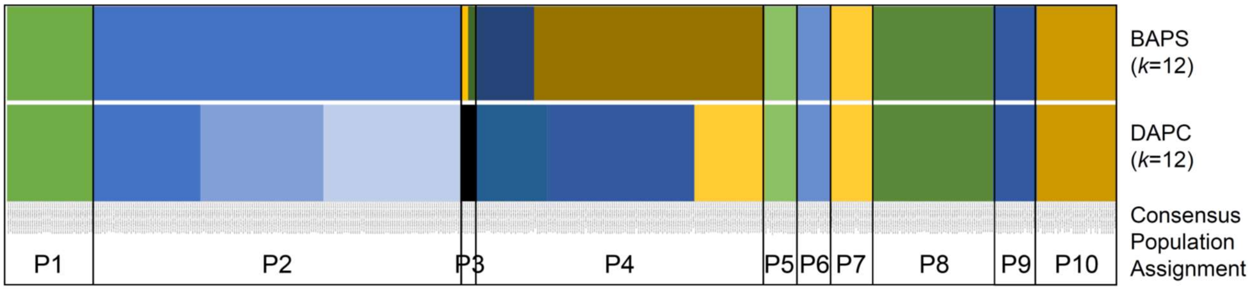

Supplementary Figure S2. Correspondence between hierBAPS and DAPC population assignment. Membership coefficients (Y-axis) of individuals (X-axis) between hierBAPS (top panel) and DAPC (middle panel). Most population assignments were in agreement between methods with the exception of heirBAPS populations 2, 5, and 6. heirBAPS populations 2 was separated into 3 DAPCpopulations. heirBAPS populations 5 and 6 had contained the same isolates as DAPC populations 6 and 7 but were assigned differently across the 2 populations. The consensus population between heirBAPS and DAPC are labeled on the bottom panel.
