# Supplementary material for: Population Genomic Analysis of Listeria monocytogenes From Food Reveals Substrate-Specific Genome Variation
Source: Front Microbiol. 2021 Feb 9;12:620033. doi: 10.3389/fmicb.2021.620033 (PMC7902062; doi:10.3389/fmicb.2021.620033)
Supplement: Supplementary file 3 [file Image_3.PDF]

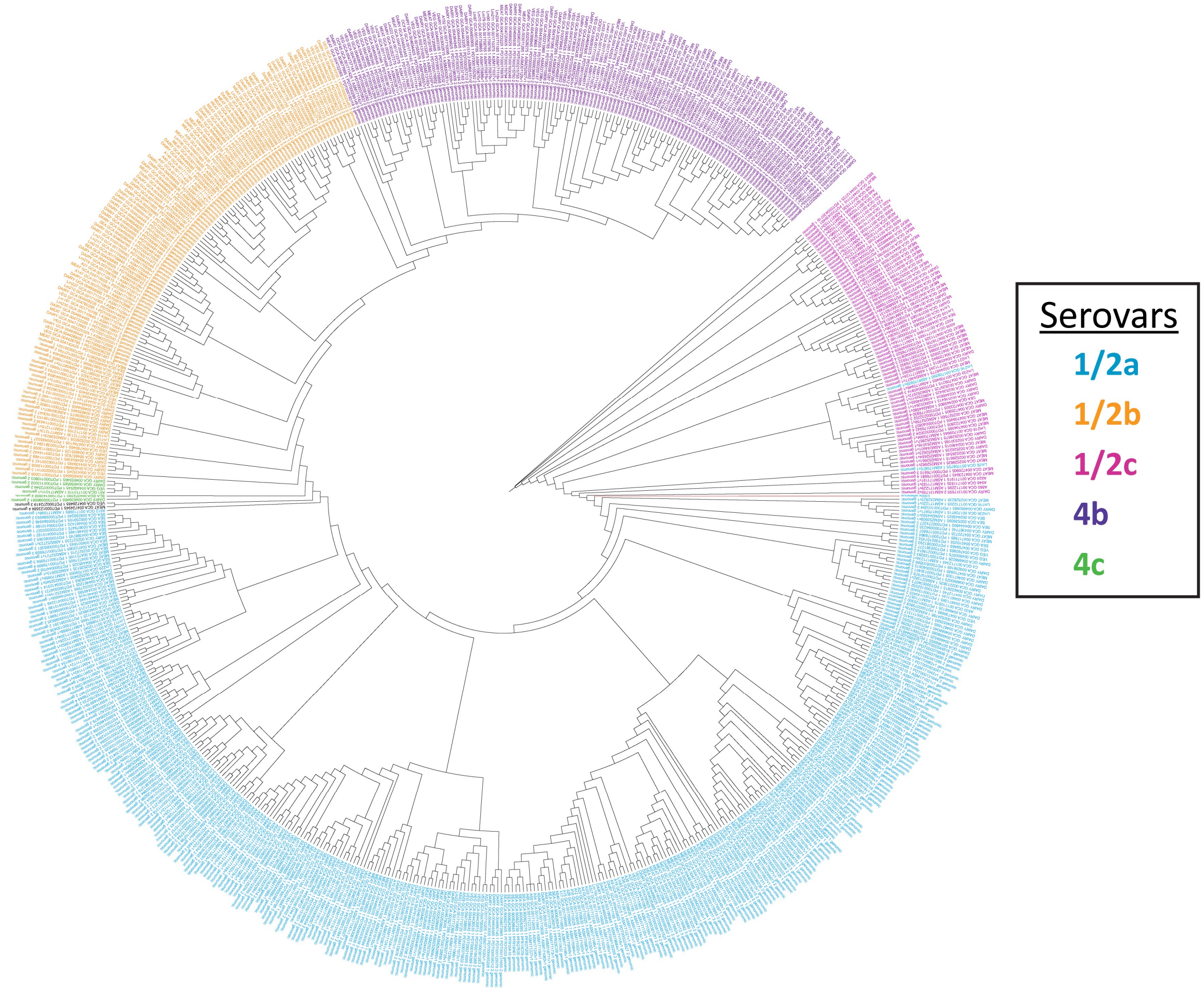

Supplementary Figure S3. Phylogenetic relationship between 504 food isolates and 167 serotyped isolates from Hingston *et al.* 2017. FastTree2 approximately-maximum likelihood phylogenetic tree inferred from a SNP alignment generated by PhaME using with 100 bootstrap replicates. Taxon labels are colored according to predicted serovar.
