# Supplementary material for: Population Genomic Analysis of Listeria monocytogenes From Food Reveals Substrate-Specific Genome Variation
Source: Front Microbiol. 2021 Feb 9;12:620033. doi: 10.3389/fmicb.2021.620033 (PMC7902062; doi:10.3389/fmicb.2021.620033)
Supplement: Supplementary file 5 [file Image_5.PDF]

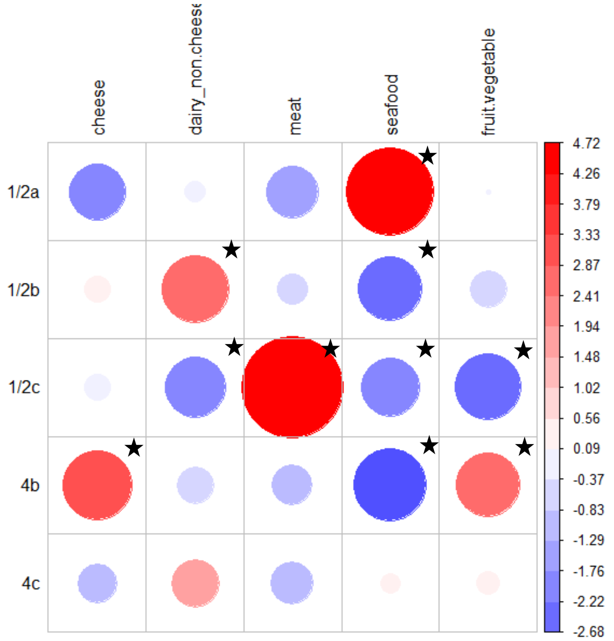

Supplementary Figure S5. Overrepresentation and underrepresentation of isolates from serotype versus food type. Plots represent standardized residuals from X^2 analysis. Circle size represents absolute value of the standardized residual, while color represents positive (red) and negative (blue) values. Stars indicate standardized residuals with absolute values ≥ 2 (two standard deviations).
