# Supplementary material for: Population Genomic Analysis of Listeria monocytogenes From Food Reveals Substrate-Specific Genome Variation
Source: Front Microbiol. 2021 Feb 9;12:620033. doi: 10.3389/fmicb.2021.620033 (PMC7902062; doi:10.3389/fmicb.2021.620033)
Supplement: Supplementary file 7 [file Table_2.DOCX]

**Supplementary Table S2. BLAST results for the 40 genes with an average BSR score ≥ 0.90 in P1 and P2 and ≤ 0.20 in P3-P10.**

| **LS-BSR Identifier** | **Description** | **Max Score** | **Total Score** | **Query Cover** | **E value** | **Per. Ident** | **Accession** |
| --- | --- | --- | --- | --- | --- | --- | --- |
| AAAHJR010000045.1_23 | Crp/Fnr family transcriptional regulator [Listeria monocytogenes] | 280 | 280 | 99% | 1.00E-96 | 100.00% | EAD2945943.1 |
| AAAIPR010000005.1_66 | DUF5011 domain-containing protein [Listeria monocytogenes] | 809 | 809 | 99% | 0 | 100.00% | WP_046811178.1 |
| AAAMDE010000052.1_1 | hypothetical protein [Listeria monocytogenes] | 285 | 285 | 99% | 5.00E-99 | 100.00% | EAD4944958.1 |
| AAATDP010000008.1_76 | hypothetical protein LMIV_0163 [Listeria monocytogenes FSL J1-208] | 65.1 | 65.1 | 97% | 2.00E-15 | 100.00% | EHY64125.1 |
| AAAXWJ010000001.1_181 | hypothetical protein [Listeria monocytogenes] | 179 | 179 | 98% | 1.00E-58 | 97.75% | WP_070265834.1 |
| AAAZDF010000008.1_24 | hypothetical protein DYZ86_00450 [Listeria monocytogenes] | 426 | 426 | 99% | 9.00E-153 | 99.55% | RKA28946.1 |
| AAAZDF010000041.1_1 | GntR family transcriptional regulator [Listeria monocytogenes] | 183 | 183 | 99% | 9.00E-60 | 100.00% | EAF8543346.1 |
| AAAZDG010000003.1_80 | TPA: GNAT family N-acetyltransferase [Listeria monocytogenes] | 89 | 89 | 97% | 4.00E-24 | 100.00% | HAK1127917.1 |
| AAAZDG010000003.1_81 | GNAT family N-acetyltransferase [Listeria monocytogenes] | 223 | 223 | 99% | 3.00E-75 | 100.00% | WP_009929307.1 |
| AABBMG010000081.1_2 | TetR/AcrR family transcriptional regulator [Listeria monocytogenes] | 332 | 332 | 99% | 2.00E-117 | 100.00% | EAG3370190.1 |
| AABDOP010000099.1_9 | TPA: leucine-rich repeat domain-containing protein [Listeria monocytogenes] | 149 | 149 | 78% | 2.00E-46 | 100.00% | HAK1046252.1 |
| AABDWF010000001.1_1140 | permease [Listeria monocytogenes] | 437 | 437 | 99% | 4.00E-154 | 100.00% | EAC8754747.1 |
| AABDZX010000005.1_151 | MULTISPECIES: BH0509 family protein [Bacteria] | 90.5 | 90.5 | 97% | 3.00E-25 | 100.00% | WP_003760374.1 |
| AABECL010000009.1_18 | hypothetical protein [Listeria monocytogenes] | 146 | 146 | 98% | 3.00E-46 | 97.14% | EAC3424073.1 |
| AABGOI010000003.1_76 | GNAT family N-acetyltransferase [Listeria monocytogenes] | 236 | 236 | 99% | 3.00E-80 | 100.00% | EFQ8062677.1 |
| AABGOR010000007.1_53 | GNAT family N-acetyltransferase [Listeria monocytogenes] | 271 | 271 | 99% | 6.00E-94 | 100.00% | WP_033918362.1 |
| AABGOV010000077.1_1 | GntR family transcriptional regulator [Listeria monocytogenes] | 150 | 150 | 98% | 4.00E-47 | 100.00% | EAF8543346.1 |
| AALWVM010000074.1_1 | LPXTG cell wall anchor domain-containing protein [Listeria monocytogenes] | 1424 | 1424 | 99% | 0 | 100.00% | EAG6470882.1 |
| AANEJE010000210.1_2 | TetR/AcrR family transcriptional regulator [Listeria monocytogenes] | 242 | 242 | 99% | 4.00E-83 | 100.00% | EAG7099462.1 |
| AANEJE010000272.1_1 | hypothetical protein [Listeria monocytogenes] | 210 | 210 | 99% | 3.00E-70 | 100.00% | EAH4421055.1 |
| CP007600.2_1131 | hypothetical protein [Listeria monocytogenes] | 193 | 193 | 89% | 4.00E-64 | 100.00% | EAG8253804.1 |
| CP007600.2_1143 | GNAT family N-acetyltransferase [Listeria monocytogenes] | 319 | 319 | 99% | 2.00E-112 | 100.00% | WP_003724679.1 |
| CP007600.2_1144 | hypothetical protein [Listeria monocytogenes] | 199 | 199 | 98% | 9.00E-67 | 100.00% | WP_003724680.1 |
| CP007600.2_1248 | hypothetical protein [Listeria monocytogenes] | 289 | 289 | 99% | 2.00E-100 | 99.36% | EAD4944958.1 |
| CP007600.2_1249 | Putative Gp2 [Listeria monocytogenes str. Scott A] | 241 | 241 | 99% | 7.00E-83 | 100.00% | EGJ24779.1 |
| CP007600.2_1670 | N-acetylmuramic acid 6-phosphate etherase [Listeria monocytogenes serotype 4b str. LL195] | 590 | 590 | 99% | 0 | 100.00% | CCO64260.1 |
| CP007600.2_2036 | methyltransferase domain-containing protein [Listeria monocytogenes] | 483 | 483 | 99% | 5.00E-174 | 100.00% | WP_003726456.1 |
| CP007600.2_2037 | TetR/AcrR family transcriptional regulator [Listeria monocytogenes] | 406 | 406 | 99% | 1.00E-145 | 100.00% | WP_003726457.1 |
| CP007600.2_2337 | TPA: Crp/Fnr family transcriptional regulator [Listeria monocytogenes] | 381 | 381 | 99% | 3.00E-135 | 99.04% | HAB8085882.1 |
| CP007600.2_2615 | LPXTG cell wall anchor domain-containing protein [Listeria monocytogenes] | 903 | 903 | 95% | 0 | 99.57% | EAF5842043.1 |
| CP007600.2_408 | hypothetical protein LMOf2365_0410 [Listeria monocytogenes serotype 4b str. F2365] | 65.1 | 65.1 | 97% | 2.00E-15 | 100.00% | AAT03195.1 |
| CP007600.2_409 | hypothetical protein LMOf2365_0411 [Listeria monocytogenes serotype 4b str. F2365] | 63.5 | 63.5 | 97% | 7.00E-15 | 96.97% | AAT03196.1 |
| CP007600.2_410 | hypothetical protein LMOf2365_0412 [Listeria monocytogenes serotype 4b str. F2365] | 66.2 | 66.2 | 97% | 7.00E-16 | 100.00% | AAT03197.1 |
| CP007600.2_416 | BH0509 family protein [Listeria monocytogenes] | 90.1 | 90.1 | 97% | 5.00E-25 | 100.00% | WP_003724329.1 |
| CP007600.2_678 | MucBP domain-containing protein [Listeria monocytogenes] | 987 | 987 | 99% | 0 | 100.00% | WP_003724403.1 |
| NXXK01000029.1_1 | TPA: LapB repeat-containing protein [Listeria monocytogenes] | 1152 | 1409 | 97% | 0 | 99.83% | HAC2392881.1 |
| NXYV01000069.1_2 | ATP-binding cassette domain-containing protein [Listeria monocytogenes] | 85.5 | 85.5 | 88% | 5.00E-23 | 95.45% | EAD4404015.1 |
| PVVE01000001.1_174 | hypothetical protein M640_00905 [Listeria monocytogenes] | 342 | 342 | 99% | 3.00E-121 | 100.00% | AGR18564.1 |
| PVVE01000001.1_175 | TPA: cell surface protein [Listeria monocytogenes] | 349 | 424 | 99% | 3.00E-122 | 100.00% | HAA6941448.1 |
| QYIA01000002.1_306 | bacteriocin immunity protein [Listeria monocytogenes] | 135 | 135 | 98% | 8.00E-41 | 100.00% | EAH3882395.1 |
