# Supplementary material for: Population Genomic Analysis of Listeria monocytogenes From Food Reveals Substrate-Specific Genome Variation
Source: Front Microbiol. 2021 Feb 9;12:620033. doi: 10.3389/fmicb.2021.620033 (PMC7902062; doi:10.3389/fmicb.2021.620033)
Supplement: Supplementary file 8 [file Table_3.DOCX]

**Supplementary Table S3. BLAST results for the 41 genes present in P1 and P2 and absent in P3-P10 using Roary.**

| **Roary ID** | **BLAST result** | **Max Score** | **Total Score** | **Query Cover** | **E- value** | **% Identity** | **Accession** |
| --- | --- | --- | --- | --- | --- | --- | --- |
| yknY_2 | ABC transporter ATP-binding protein | 418.00 | 418.00 | 0.99 | 0.00 | 0.99 | [EFT8853371.1](https://www.ncbi.nlm.nih.gov/protein/EFT8853371.1?report=genbank&log$=prottop&blast_rank=1&RID=ZYHPEWM001R) |
| fepC | ABC transporter ATP-binding protein | 527.00 | 527.00 | 0.99 | 0.00 | 1.00 | [WP_003724600.1](https://www.ncbi.nlm.nih.gov/protein/WP_003724600.1?report=genbank&log$=prottop&blast_rank=1&RID=ZYHPEWM001R) |
| yknZ_2 | ABC transporter permease | 710.00 | 710.00 | 0.99 | 0.00 | 1.00 | WP_003724328.1 |
| group_7597 | Acetylglutamate kinase | 497.00 | 497.00 | 0.99 | 0.00 | 1.00 | [CCO64188.1](https://www.ncbi.nlm.nih.gov/protein/CCO64188.1?report=genbank&log$=prottop&blast_rank=1&RID=ZYHPEWM001R) |
| group_7596 | acetylornithine transaminase | 785.00 | 785.00 | 0.99 | 0.00 | 1.00 | [WP_003725999.1](https://www.ncbi.nlm.nih.gov/protein/WP_003725999.1?report=genbank&log$=prottop&blast_rank=1&RID=ZYHPEWM001R) |
| group_640 | actin assembly-inducing protein ActA | 989.00 | 989.00 | 0.99 | 0.00 | 1.00 | [WP_077952120.1](https://www.ncbi.nlm.nih.gov/protein/WP_077952120.1?report=genbank&log$=prottop&blast_rank=1&RID=ZYHPEWM001R) |
| group_7568 | alpha-ribazole phosphatase | 368.00 | 368.00 | 0.99 | 0.00 | 0.99 | [WP_003724691.1](https://www.ncbi.nlm.nih.gov/protein/WP_003724691.1?report=genbank&log$=prottop&blast_rank=1&RID=ZYHPEWM001R) |
| group_6401 | baseplate J/gp47 family protein | 776.00 | 776.00 | 0.99 | 0.00 | 1.00 | [WP_096827102.1](https://www.ncbi.nlm.nih.gov/protein/WP_096827102.1?report=genbank&log$=prottop&blast_rank=1&RID=ZYHPEWM001R) |
| group_7486 | BH0509 family protein | 90.10 | 90.10 | 0.97 | 0.00 | 1.00 | [WP_003724329.1](https://www.ncbi.nlm.nih.gov/protein/WP_003724329.1?report=genbank&log$=prottop&blast_rank=1&RID=ZYHPEWM001R) |
| ubiG_1 | class I SAM-dependent methyltransferase | 514.00 | 514.00 | 0.99 | 0.00 | 1.00 | [WP_003726668.1](https://www.ncbi.nlm.nih.gov/protein/WP_003726668.1?report=genbank&log$=prottop&blast_rank=1&RID=ZYHPEWM001R) |
| group_7646 | ComF family protein | 422.00 | 422.00 | 0.99 | 0.00 | 1.00 | [WP_003726225.1](https://www.ncbi.nlm.nih.gov/protein/WP_003726225.1?report=genbank&log$=prottop&blast_rank=1&RID=ZYHPEWM001R) |
| group_7647 | DEAD/DEAH box helicase | 890.00 | 890.00 | 0.99 | 0.00 | 1.00 | [WP_003726226.1](https://www.ncbi.nlm.nih.gov/protein/WP_003726226.1?report=genbank&log$=prottop&blast_rank=3&RID=ZYHPEWM001R) |
| group_5617 | DNA topology modulation protein | 313.00 | 313.00 | 0.99 | 0.00 | 0.99 | [EAF8511836.1](https://www.ncbi.nlm.nih.gov/protein/EAF8511836.1?report=genbank&log$=prottop&blast_rank=1&RID=ZYHPEWM001R) |
| group_7540 | DNA-3-methyladenine glycosylase | 429.00 | 429.00 | 0.99 | 0.00 | 1.00 | [WP_003724850.1](https://www.ncbi.nlm.nih.gov/protein/WP_003724850.1?report=genbank&log$=prottop&blast_rank=1&RID=ZYHPEWM001R) |
| group_6408 | DUF1073 domain-containing protein | 953.00 | 953.00 | 0.99 | 0.00 | 1.00 | [WP_096827068.1](https://www.ncbi.nlm.nih.gov/protein/WP_096827068.1?report=genbank&log$=prottop&blast_rank=1&RID=ZYHPEWM001R) |
| group_6402 | DUF2634 domain-containing protein | 241.00 | 241.00 | 0.99 | 0.00 | 1.00 | [WP_096827100.1](https://www.ncbi.nlm.nih.gov/protein/WP_096827100.1?report=genbank&log$=prottop&blast_rank=1&RID=ZYHPEWM001R) |
| group_5846 | DUF3899 domain-containing protein | 223.00 | 223.00 | 0.99 | 0.00 | 1.00 | [WP_003724613.1](https://www.ncbi.nlm.nih.gov/protein/WP_003724613.1?report=genbank&log$=prottop&blast_rank=1&RID=ZYHPEWM001R) |
| group_5661 | DUF4352 domain-containing protein | 327.00 | 327.00 | 0.86 | 0.00 | 1.00 | [EAF4691877.1](https://www.ncbi.nlm.nih.gov/protein/EAF4691877.1?report=genbank&log$=prottop&blast_rank=2&RID=ZYHPEWM001R) |
| group_7505 | DUF5067 domain-containing protein | 281.00 | 281.00 | 0.99 | 0.00 | 0.99 | [EAF6296160.1](https://www.ncbi.nlm.nih.gov/protein/EAF6296160.1?report=genbank&log$=prottop&blast_rank=1&RID=ZYHPEWM001R) |
| group_7639 | fumarylacetoacetate hydrolase family protein | 575.00 | 575.00 | 0.99 | 0.00 | 1.00 | [WP_003726824.1](https://www.ncbi.nlm.nih.gov/protein/WP_003726824.1?report=genbank&log$=prottop&blast_rank=1&RID=ZYHPEWM001R) |
| group_7629 | GNAT family N-acetyltransferase | 317.00 | 317.00 | 0.99 | 0.00 | 1.00 | [WP_003724616.1](https://www.ncbi.nlm.nih.gov/protein/WP_003724616.1?report=genbank&log$=prottop&blast_rank=1&RID=ZYHPEWM001R) |
| group_3036 | helicase-exonuclease AddAB subunit AddB | 2366.00 | 2366.00 | 0.99 | 0.00 | 1.00 | ECH3376517.1 |
| group_3638 | helicase-exonuclease AddAB subunit AddB | 2500.00 | 2500.00 | 0.99 | 0.00 | 1.00 | [EGF7306213.1](https://www.ncbi.nlm.nih.gov/protein/EGF7306213.1?report=genbank&log$=prottop&blast_rank=1&RID=ZYHPEWM001R) |
| group_5827 | HTH-type transcriptional repressor GlcR | 514.00 | 514.00 | 0.99 | 0.00 | 1.00 | [RJY61335.1](https://www.ncbi.nlm.nih.gov/protein/RJY61335.1?report=genbank&log$=prottop&blast_rank=2&RID=ZYHPEWM001R) |
| group_6403 | hypothetical protein | 221.00 | 221.00 | 0.98 | 0.00 | 0.98 | [EAG8216203.1](https://www.ncbi.nlm.nih.gov/protein/EAG8216203.1?report=genbank&log$=prottop&blast_rank=3&RID=ZYHPEWM001R) |
| group_6404 | hypothetical protein | 548.00 | 548.00 | 0.98 | 0.00 | 1.00 | [WP_096827117.1](https://www.ncbi.nlm.nih.gov/protein/WP_096827117.1?report=genbank&log$=prottop&blast_rank=2&RID=ZYHPEWM001R) |
| group_6405 | hypothetical protein | 256.00 | 256.00 | 0.99 | 0.00 | 1.00 | [WP_096827083.1](https://www.ncbi.nlm.nih.gov/protein/WP_096827083.1?report=genbank&log$=prottop&blast_rank=1&RID=ZYHPEWM001R) |
| group_6406 | hypothetical protein | 408.00 | 408.00 | 0.99 | 0.00 | 1.00 | [WP_096827081.1](https://www.ncbi.nlm.nih.gov/protein/WP_096827081.1?report=genbank&log$=prottop&blast_rank=1&RID=ZYHPEWM001R) |
| group_6407 | hypothetical protein | 301.00 | 301.00 | 0.99 | 0.00 | 1.00 | [WP_096827073.1](https://www.ncbi.nlm.nih.gov/protein/WP_096827073.1?report=genbank&log$=prottop&blast_rank=1&RID=ZYHPEWM001R) |
| group_7504 | hypothetical protein | 156.00 | 156.00 | 0.98 | 0.00 | 1.00 | [WP_003724358.1](https://www.ncbi.nlm.nih.gov/protein/WP_003724358.1?report=genbank&log$=prottop&blast_rank=2&RID=ZYHPEWM001R) |
| group_7618 | hypothetical protein | 333.00 | 333.00 | 0.99 | 0.00 | 1.00 | [GAM92250.1](https://www.ncbi.nlm.nih.gov/protein/GAM92250.1?report=genbank&log$=prottop&blast_rank=1&RID=ZYHPEWM001R) |
| group_4619 | hypothetical protein M637_12820 | 639.00 | 639.00 | 0.99 | 0.00 | 1.00 | [AGR06437.1](https://www.ncbi.nlm.nih.gov/protein/AGR06437.1?report=genbank&log$=prottop&blast_rank=1&RID=ZYHPEWM001R) |
| group_4765 | lactococcin 972 family bacteriocin | 270.00 | 270.00 | 0.99 | 0.00 | 1.00 | [WP_003725327.1](https://www.ncbi.nlm.nih.gov/protein/WP_003725327.1?report=genbank&log$=prottop&blast_rank=1&RID=ZYHPEWM001R) |
| group_3967 | LPXTG cell wall anchor domain-containing protein | 1163.00 | 1163.00 | 0.99 | 0.00 | 1.00 | [EAF9429219.1](https://www.ncbi.nlm.nih.gov/protein/EAF9429219.1?report=genbank&log$=prottop&blast_rank=5&RID=ZYHPEWM001R) |
| group_4309 | LPXTG cell wall anchor domain-containing protein | 971.00 | 971.00 | 0.99 | 0.00 | 0.99 | EAG8879194.1 |
| group_5936 | LPXTG cell wall anchor domain-containing protein | 903.00 | 903.00 | 0.95 | 0.00 | 1.00 | [EAF5842043.1](https://www.ncbi.nlm.nih.gov/protein/EAF5842043.1?report=genbank&log$=prottop&blast_rank=2&RID=ZYHPEWM001R) |
| group_7548 | LytTR family transcriptional regulator | 269.00 | 269.00 | 0.99 | 0.00 | 1.00 | [WP_003726676.1](https://www.ncbi.nlm.nih.gov/protein/WP_003726676.1?report=genbank&log$=prottop&blast_rank=1&RID=ZYHPEWM001R) |
| group_5464 | membrane protein | 405.00 | 405.00 | 0.99 | 0.00 | 1.00 | [AGR06920.1](https://www.ncbi.nlm.nih.gov/protein/AGR06920.1?report=genbank&log$=prottop&blast_rank=1&RID=ZYHPEWM001R) |
| group_7584 | N-acetyltransferase | 342.00 | 342.00 | 0.99 | 0.00 | 0.99 | [GAM93812.1](https://www.ncbi.nlm.nih.gov/protein/GAM93812.1?report=genbank&log$=prottop&blast_rank=1&RID=ZYHPEWM001R) |
| group_6409 | phage terminase large subunit | 969.00 | 969.00 | 0.97 | 0.00 | 1.00 | [WP_096827115.1](https://www.ncbi.nlm.nih.gov/protein/WP_096827115.1?report=genbank&log$=prottop&blast_rank=4&RID=ZYHPEWM001R) |
| group_7501 | phosphoribosyl-ATP diphosphatase | 205.00 | 205.00 | 0.99 | 0.00 | 1.00 | [WP_003725463.1](https://www.ncbi.nlm.nih.gov/protein/WP_003725463.1?report=genbank&log$=prottop&blast_rank=1&RID=ZYHPEWM001R) |
| group_7599 | phosphotransferase family protein | 543.00 | 543.00 | 0.99 | 0.00 | 1.00 | [WP_003726713.1](https://www.ncbi.nlm.nih.gov/protein/WP_003726713.1?report=genbank&log$=prottop&blast_rank=1&RID=ZYHPEWM001R) |
| group_7482 | Pyrroline-5-carboxylate reductase | 529.00 | 529.00 | 0.99 | 0.00 | 1.00 | [WP_003724318.1](https://www.ncbi.nlm.nih.gov/protein/WP_003724318.1?report=genbank&log$=prottop&blast_rank=2&RID=ZYHPEWM001R) |
| group_3521 | Ribonuclease, Rne/Rng family | 924.00 | 924.00 | 0.99 | 0.00 | 1.00 | CCO64142.1 |
| group_7536 | RidA family protein | 228.00 | 228.00 | 0.90 | 0.00 | 1.00 | [WP_003724775.1](https://www.ncbi.nlm.nih.gov/protein/WP_003724775.1?report=genbank&log$=prottop&blast_rank=1&RID=ZYHPEWM001R) |
| group_5573 | tetratricopeptide repeat protein | 315.00 | 315.00 | 0.99 | 0.00 | 1.00 | [WP_003726184.1](https://www.ncbi.nlm.nih.gov/protein/WP_003726184.1?report=genbank&log$=prottop&blast_rank=2&RID=ZYHPEWM001R) |
| group_5586 | ThiF family adenylyltransferase | 692.00 | 692.00 | 0.99 | 0.00 | 1.00 | [WP_003727025.1](https://www.ncbi.nlm.nih.gov/protein/WP_003727025.1?report=genbank&log$=prottop&blast_rank=1&RID=ZYHPEWM001R) |
| group_5880 | thioredoxin family protein | 194.00 | 194.00 | 0.98 | 0.00 | 0.99 | [WP_003725609.1](https://www.ncbi.nlm.nih.gov/protein/WP_003725609.1?report=genbank&log$=prottop&blast_rank=2&RID=ZYHPEWM001R) |
| group_7576 | tRNA (adenosine(37)-N6)-dimethylallyltransferase MiaA | 602.00 | 602.00 | 0.99 | 0.00 | 1.00 | [WP_003726658.1](https://www.ncbi.nlm.nih.gov/protein/WP_003726658.1?report=genbank&log$=prottop&blast_rank=1&RID=ZYHPEWM001R) |
| group_6400 | Uncharacterised protein | 84.70 | 84.70 | 0.97 | 0.00 | 1.00 | [CWU07660.1](https://www.ncbi.nlm.nih.gov/protein/CWU07660.1?report=genbank&log$=prottop&blast_rank=1&RID=ZYHPEWM001R) |
| group_7594 | uroporphyrinogen-III synthase | 487.00 | 487.00 | 0.99 | 0.00 | 1.00 | [WP_003725682.1](https://www.ncbi.nlm.nih.gov/protein/WP_003725682.1?report=genbank&log$=prottop&blast_rank=1&RID=ZYHPEWM001R) |
